# CME providers’ experiences and practices in Pakistan: a case study

**DOI:** 10.1186/s12909-024-05201-y

**Published:** 2024-03-12

**Authors:** Farhan Saeed Vakani, Apo Demirkol, Kerry Uebel, Chinthaka Balasooriya

**Affiliations:** 1 Dow Institute of Health Professionals Education, Dow University of Health Sciences, Baba-e-Urdu Road, 74200 Karachi, Pakistan; 2 Drug and Alcohol Services, SESLHD, Prince of Wales Hospital Pain Management Centre, School of Population Health, University of New South Wales, Sydney, Australia; 3 School of Population Health, University of New South Wales, 2052 Sydney, NSW Australia

**Keywords:** CME providers, Recommendations, Physicians, Pakistan

## Abstract

**Background:**

Pakistan has made numerous attempts to establish and implement a national mandatory CME program which currently do not exist. The purpose of this study is to explore the views of major CME providers in order to identify possible strengths and weaknesses in the current program, and offer evidence-based recommendations to help further enhance the national CME program in Pakistan.

**Methods:**

An exploratory study design using a case study approach through in depth interviews was conducted to examine CME providers’ experiences and perceptions. The study was conducted in Pakistan between August and November 2019 with CME providers from Sindh, Punjab, the North-West Frontier Province, and the Federal Capital Territory. Thirty-six providers recognised by the Pakistan Medical and Dental Council who were involved in providing CME activities at the national level and whose contact information was publicly available on their websites, were selected for the study. Of the 36 providers invited, 22 participated in this study.

**Results:**

The results generated several organising themes grouped into three major themes: (1) CME current practices, (2) CME past experiences, and (3) Future developments.

**Conclusion:**

Participants recommended needs-based educational activities for physicians, a well-structured central regulatory CME body collaborating with existing providers, involving experienced providers for rural CME, accrediting diverse local providers, limiting commercial entities’ role, and implementing CME with proper preparation and a phased approach.

## Background

An early definition of CME, as stated by the American Medical Association (AMA), emphasises that it comprises educational activities aimed at maintaining, developing, or enhancing a physician’s knowledge, skills, professional performance, and relationships, ultimately benefiting patients, the public, and the medical profession as a whole [1]. This definition underscores the importance of continuing education in maintaining physicians’ competence and delivering high-quality healthcare through active learning programs [2].

The concept of mandatory CME originated in North America and was later introduced to Europe and other countries [3–6]. However, implementing mandatory CME programs has proven challenging in most developing nations [2, 7]. India, for instance, initially faced difficulties when introducing a national CME system in 2002 through the Medical Council of India (MCI), and steady progress has been made over the years through state medical councils [6, 8]. Similarly, China began the process in 1996 but did not fully implement it until 2001. The Indonesian government initiated a national CME program in 2006 but with a shortened deadline for rapid implementation, resulting in quality and compliance concerns [6]. Consequently, the World Health Organization (WHO) standardised CME practices in the region and published SEAR regional guidelines for CME/CPD activities in 2010 [7, 9].

### Context and aims of study

In Pakistan, various institutions such as medical universities, medical colleges, medical professional associations, the College of Physicians and Surgeons Pakistan (CPSP), and the Pakistan Medical and Dental Associations (PMA & PDA) have been providing voluntary CME activities in Pakistan for many years [10, 11].

Over this time, several attempts have been made to establish a mandatory national CME program, but such a system does not yet exist [11–13]. It is however noted that these efforts to establish a mandatory program has led to some significant developments in CME more broadly.

Much can be learnt by reflecting on the experience of attempting to introduce a mandatory CME program in Pakistan. Work by Thahim (2015) suggests that one of the key reasons for limited success may be that key institutions in Pakistan have not accepted or shared the responsibility for implementing mandatory CME [11]. Other factors that have hindered the introduction of mandatory CME include the lack of regulatory and technical support and the absence of a CME unit at the Pakistan Medical and Dental Council (PM&DC) [11, 12]. Further exploration of the reasons noted above and extending the exploration to include the perspectives of CME providers, can be very valuable in shaping the future of CME in Pakistan.

This study aimed to involve major CME providers in Pakistan to explore their experiences and perspectives on the current state of CME in the country [14, 15]. This exploration was designed to identify strengths and potential gaps in CME provision, which could inform the development of recommendations for the future development of CME in Pakistan.

## Methods and participants

An exploratory study using a case study approach was conducted [16, 17], with Pakistan chosen as a low-middle income country that could offer insights for other countries in South-East Asia (SEAR) and Eastern Mediterranean (EMR) region. We used in-depth interviews [17–19] to examine the experiences and perceptions of CME providers operating in Pakistan, recognised by the Pakistan Medical and Dental Council (PM&DC) [14, 15]. These national and regional organizations were accredited by the Council based on the inclusion in the council’s list, performance and involvement in CME/CPD provision and registered with the office of the registration societies [13].

A total of 36 providers involved in national-level CME activities were selected for the study, of which 30 initially agreed to participate. Eventually, 22 providers took part (See Table 1). Ethical approval was obtained from the University of New South Wales (HC180960) and Hamdard University (ERB1904).

**Table 1:** CME Providers and participant’s profile

S. No	CME Provider	Province/Region	Participant Role
1)	Pakistan Dental Association	Sindh	Administration
2)	Pakistan Medical Association	Sindh	Administration
3)	University of Health Sciences	Punjab	Professor of Dentistry
4)	Riphah International University	Federal Capital	Professor of Dentistry
5)	Gandhara University	NWFP	Professor of Dentistry
6)	Ziauddin University	Sindh	Medical educationist
7)	University of Lahore	Punjab	Medical educationist
8)	Shifa College of Medicine	Federal Capital	Medical educationist
9)	Armed Forces PGMI	Federal Capital	Medical educationist
10)	Dow University of Health Sciences	Sindh	Medical educationist
11)	Khyber University	NWFP	Medical educationist
12)	Pakistan Society of Neurology	Sindh	Professor of Medicine
13)	Indus Hospital	Sindh	Professor of Medicine
14)	Pakistan Society for the Study of Liver Disease	Sindh	Professor of Medicine
15)	Shaukat Khanum Cancer Hospital	Punjab	Professor of Oncology
16)	PGMI Peshawar	NWFP	Professor of Oncology
17)	Isra University, Al-Nafees Medical College	Federal Capital	Administration
18)	College of Family Medicine	Sindh	Specialist College
19)	College of Physicians & Surgeons Pakistan	Sindh	Specialist College
20)	King Edward University	Punjab	Professor of Surgery
21)	Liaquat University	Sindh	Professor of Surgery
22)	Aga Khan University	Sindh	Administration

The interviews, conducted between August 2019 and November 2019 by FV, were held at the providers’ premises, in English language. An in-depth semi-structured interview guide was used, and participants provided informed consent and agreed for the interviews to be recorded. The interviews lasted approximately 30 to 40 min and were audio-recorded and transcribed in full. Participants were assured that only the interviewer and research team would have access to their interview recordings, that their identities would remain confidential, and findings would only be shared in de-identified form. The findings were validated by sharing with the participants to ensure accuracy and alignment with their experiences.

A total of 22 participants (*n* = 22) were interviewed, with each individual providing insight into their respective institutional CME department (See Table 1). The participants were geographically distributed across Pakistan, which consists of four provinces and one federal territory. Specifically, 11 participants were interviewed from Sindh province, four from Punjab, three from the North-West Frontier Province, and four from the Federal Capital Territory. Baluchistan was not included in the study as no providers were listed from that region. Among the participants, 14 out of 22 were male academics. The age range of the participants was between 50 and 65 years.

**Fig. 1:**
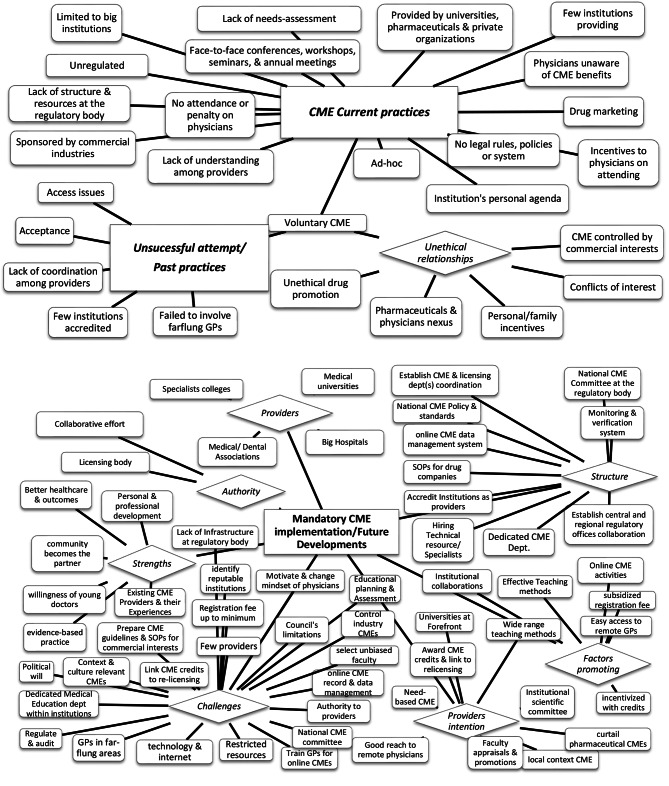
Thematic network technique

Data analysis involved three independent reviewers (FV, AD, and KU). An iterative process was followed, with reviewers reading and analysing the transcripts. Content analysis was used to code the interview transcripts, starting from basic themes and organizing them into higher-level organizing themes. Thematic networks technique [20], using web-like illustrations, was employed (See Fig. 1). The themes identified by each reviewer were compared in group meetings to establish a final list of agreed-upon themes.

## Results

**Fig. 2:**
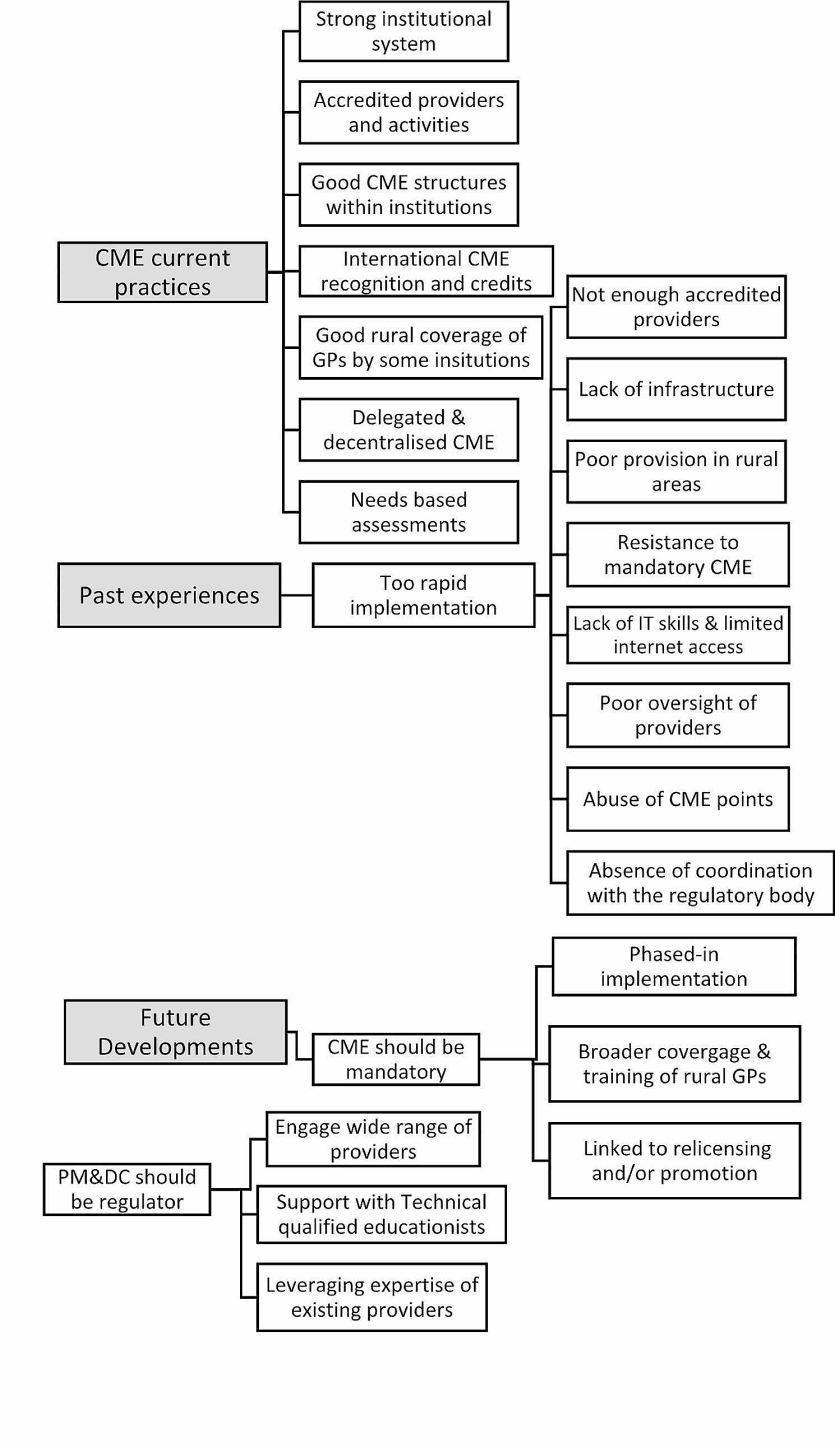
Themes and subthemes

The results generated several organising themes grouped into three major themes: (1) CME current practices, (2) CME past experiences, and (3) Future developments (See Fig. 2). Each global theme is described below in detail. The text in quotation represents quotes from the interviews.

### CME current practices

During the exploration of current CME practices, several strengths were identified and grouped into organising themes. These themes included a strong institutional system, accredited providers and activities, and international recognition of CME and credits.

The participants in the study represented various CME providers, including universities, medical colleges, postgraduate specialist training institutions, and physician medical associations. Most participants expressed that the existence of a robust CME structure was the result of previous efforts to implement mandatory CME. These providers had received accreditation from the national council and were actively offering CME credits. Additionally, some interviewees mentioned that their organizations focused on providing needs-based activities, which involved a systematic process for the delivery of CME. A Professor of surgery spoke on the importance of local data collection and literature review when identifying learners’ needs and commented:

“When CME proposes or recommends a certain line of activity, then it has to be based, not simply on, the individual’s personal opinion, but it should be backed by strong evidence.” (Participant # 13).

A representative at a specialist college stated:

“Our CME programs are planned mainly according to the needs of the target audience.” (Participant # 19).

Among the CME providers, four major institutions stood out for having a robust system that ensured sufficient coverage, including rural doctors. These institutions effectively implemented CME through a decentralized mechanism and displayed confidence in their capacity to train local GPs. A study participant representing the leadership of a specialist college stated:

“Our college has a good reach to train local physicians across Pakistan and represents General Practitioners (GPs) at the most. We have been conducting CME for the last fourteen years.” (Participant # 3).

Medical Education representative at the specialist college had a different approach in mind to cater to GPs:

“We are also launching a movement for general practitioners, CME for GPs, as they constitute a major portion of our medical workforce. Our aim is to replace the structured membership training requirement for general practitioners with certain years of good standing and completion for a hundred CME *[activities (sic.)]*.” (Participant # 19).

A small number of providers’ CME and credits are recognised internationally, which allowed their physicians to benefit in the accrual of credits to maintain international licensure while attending CME in Pakistan. A Professor in Oncology shared:

“This [CME] enables them to maintain international registration intact.” (Participant # 8).

Some good institutional models encouraged physicians to attend CME for promotions and to attain privileges within the institutions. A Professor in Medicine shared his feeling:

“Most of the CME…are linked to promotion.” (Participant # 14).

### CME past experiences

During the interviews, participants highlighted various challenges that contributed to the earlier failure of implementing mandatory CME. These challenges were categorized into organizing themes, including non-standardized activities, insufficient rural coverage and training, limited number of accredited providers, rapid implementation, and lack of infrastructure.

In contrast to the earlier section where many providers mentioned the existence of strong CME structures, some participants expressed a view that nationally organized CME activities lacked standardization. This was attributed to limited attention to CME standards and a lack of needs-based educational planning:

“[CME] they are mostly done on the interest of the organisers.” (Participant #13).

The participants expressed concerns regarding the involvement of commercial interests in the activities and the potential for bias in the educational content. A Professor in Medicine commented as follows:

“Most of the CME is free of charge… lot is organised by pharmaceutical industries…and are linked to drug promotion compared to actual education.” (Participant # 14).

A medical educationist commented:

“CME activities became a sit-and-get session for a lot of people in a lot of institutions…” (Participant # 10).

Several participants noted that after graduating, many doctors received no CME, particularly those practicing in rural areas. These doctors often face challenges in taking time off and traveling long distances to attend CME events. Additionally, limited internet access and a lack of necessary computer skills were identified as potential barriers for physicians in accessing online CME resources. According to a study participant and a medical educationist:

“If we are thinking of doing something online, then there are a number of physicians who do not have the access, capacity or skills to go online and do things.” (Participant # 9).

Another participant and a faculty in dentistry from a large public sector university added:

“Absence of internet connection at some places make physicians handicapped to attend.”(Participant # 5).

Most believed that implementing compulsory CME was not successful due to insufficient numbers of accredited providers. A clinical professor expressed:

“there are only a few universities that can have activities, and they (regulatory body) were unable to accredit most societies and other organisations……” (Participant 14).

Some participants reflected that due to the limited number of providers, the physicians had to travel long distances to attend accredited activities, resulting in resistance from the physicians to participate, or even potential misuse of CME credit points and certificates. A faculty in medical education stated:

“Pharmaceutical industries started selling these certificates…even if you haven’t done a CME, they will offer you those certificates in different courses, from different conferences which they have sponsored.” (Participant # 10).

Participants also described insufficient preparation as a cause of failure in implementation:

“program was too ambitious and that was a sort of switching on suddenly”, stated by a director medical education at a specialist college. (Participant # 19)

In addition to the factors mentioned above, some participants expressed concerns about the lack of trained staff at the regulatory body, the presence of a weak central data management system, and the absence of coordination between CME providers and the regulatory body as contributing factors to the overall failure of CME implementation.

## Future developments

The participants unanimously acknowledged the necessity for thorough groundwork and preparation in implementing mandatory CME. They put forth several recommendations that can be categorized into different areas, including governance, provider accreditation, broader coverage and training for rural physicians, infrastructure and staff, phased implementation, and promotion of inter-coordination. The majority of participants shared the view that the PM&DC, being the authorized body for licensing physicians, should have exclusive authority in regulating CME. As mentioned by a participant representative at the specialist college:

“PM&DC is the body that should be legally responsible to control CME.” (Participant # 3).

Several participants emphasized the importance of engaging a wider range of providers and leveraging the expertise of existing institutions to extend the reach of CME to remote areas, specifically targeting GPs. A director of CME department believed:

“If they gather the institutions who are doing it and initially get their help to start region by region, they can do it.” (Participant # 15).

A participant commented:

“And you know my… you can call it wishful thinking that general practitioners are the ones who actually cater to 80% of the country and they make or break people, right? So, we ensure that GPs are included…” (Participant # 1).

A participant commented to target GPs especially in remote areas:

“If we educate our general practitioners, we can qualitatively improve the health care…stated by the Director of Medical Education at the Specialist College.” (Participant # 19).

Regarding the range of providers, most participants’ responses showed that the public and private medical universities, medical colleges, specialist professional colleges, mega hospitals and medical or dental associations could be included. Many participants commented on the need to limit and regulate drug companies’ role due to their vested commercial interest. An administrative representative at a medical university stated:

“One thing that should be mandated, and that has to be done through an act of parliament or ordinance or whatever legal method. It’s that; these pharmaceuticals shouldn’t be allowed to provide CME.” (Participant # 17).

Despite this, several participants acknowledge the potential for pharmaceutical sponsorship of educational activities via their unrestricted educational funds. As indicated by a participant and director in medical education:

“If they are sponsoring CME activity… it should be declared to the participants and known to all.” (Participant # 19).

Most participants suggested a vast array of CME topics using conferences, seminars, symposiums, workshops, research and routine scheduled activities. They believed that the CME events could be delivered online at locations accessible to GPs based in rural areas. A Professor in Surgery & Medical Educationist stated:

“GPs cannot…afford travelling to bigger cities, so taking advantage of the internet we could provide online activities in a town hall or some common place.” (Participant # 1).

Several participants proposed a phase-in period to prepare providers before implementing mandatory CME. A few believed that linking CME to re-licensing is quite a complex process that requires huge efforts and patience. Study participant at a specialist college commented:

“CME in Pakistan… is premature to be transformed into re-licensing in the near future… so we actually think that any change should be gradual and to make an effort slowly and progressively.” (Participant # 19).

Most participants agreed and recommended that the structures within the National CME body are important, preferring suitably qualified medical educationalist’s involvement in the central body, so that it can help guide the providers’ efforts. According to a faculty in medical education:

“We need technical people who should guide. I think that is the missing link you need technical people in PM&DC who are working there full-time and who should not change with any council and who should be full-time working in PM&DC to support all these different processes.” (Participant # 10).

## Discussion

The findings, categorized under the theme of ‘CME current practices,’ reflect a positive outlook and significant potential for further advancements in CME and lifelong learning throughout Pakistan. The interviews revealed that previous endeavours to establish CME initiatives at a national level have laid a promising foundation in accrediting providers, albeit in limited numbers. However, attempts at policy development and implementation have encountered challenges and yielded mixed results [12, 13]. There were some institutions that provided needs-based activities, which involved a systematic process for CME delivery. In her article, Shannon has identified the importance of a needs-assessment process as a foundation to plan educational activities to meet the precise learning needs of physicians [21]. A national survey by Siddiqui et al. reported that most Pakistani physicians favoured needs-based CME programs [22]. An illustrative example is of the College of Physicians and Surgeons Pakistan (CPSP), a premier postgraduate institution that potentially apply the feedback strategy through program evaluation to plan for future CME activities [23]. The CME program delivered by CPSP lends itself to further research that may be generalised to the rest of the country. Participants highlighted the presence of various institutions that incentivize physicians to participate in Continuing Medical Education (CME) by offering promotions and academic privileges within their respective establishments. This approach is not unique and can be observed in other contexts, such as Sudan, where credit points are not mandatory for relicensing but serve as a motivating factor for faculty promotion [24].

In terms of CME for GPs practicing in rural areas, study participants expressed contrasting opinions on the best approach. These divergent viewpoints have historically resulted in limited opportunities for collaboration, and this pattern of contradictory statements is not a new phenomenon [25]. The respondents identified a limited number of institutions that possess comprehensive resources to deliver CME to GPs practising in rural areas. In his report, Jooma acknowledged that these institutions have extensive experience and a wide reach among rural physicians in Pakistan [10]. In contrast to this perspective, certain interviewees held the view that the inadequate coverage of rural physicians in terms of CME is a result of certain providers not giving sufficient attention to the outreach efforts to rural areas.

Participants identified several key barriers for rural physicians which include limited time and financial resources, challenges in traveling to central venues, inadequate information technology (IT) skills among physicians, unreliable internet connectivity, and limited opportunities for participation due to a scarcity of accredited providers. Our study results mirror the findings on the perceived barriers to CPD in a study done in Scotland [26], and a recent regional study in Sri Lanka on ‘Grade Medical Officers’ [27].

Insufficient preparation and rapid implementation of a CME program in Pakistan were identified as factors contributing to its failure. Similar challenges, such as government non-acceptance or brief timeframes for CME system development, have been experienced in other developing countries like India, Indonesia, and China [6].

Some study participants expressed their concern about the sponsorship of the pharmaceutical industry in CME program delivery which is a widely recognised global problem [28]. Uncontrolled commercialism can undermine the integrity of the education programs; but limited or lack of dedicated resources may put such activities out of reach for a significant proportion of physicians practising in resource-poor countries [29]. Therefore, to provide effective CME programs and professional development of the physicians, it is recommended that an ethical contract between the commercial interest and its funding is established [30]. Most participants in the study agreed that the Pakistan Medical and Dental Council (PM&DC) should assume the role of the regulatory body for CME. They emphasized the importance of collaboration between the PM&DC and major stakeholders in establishing a national CME committee and formulating policies that could be implemented by accredited providers. Study findings highlighted the significance of involving medical educators in the central body, promoting needs-based educational activities, establishing mechanisms for monitoring CME activities, delegating administrative responsibilities to larger providers for accrediting local providers and educational events, issuing credits to participants, and fostering regional collaborations.

The limitations of this study need to be noted. The perspectives examined in this research are primarily based on the participants’ individual experiences within their specific contexts, which may not provide a comprehensive representation of the overall landscape. However, certain findings may still hold relevance and applicability to similar settings. To mitigate this limitation, we made efforts to engage with a wide range of stakeholders, aiming to present a holistic overview based on the current status of CME in Pakistan.

## Conclusion

This study examined providers’ perspectives on current practices, past experiences, and future development strategies for CME in Pakistan. The participants emphasized the importance of planning needs-based educational activities for physicians. Key findings indicate the necessity for a well-structured central regulatory body to collaborate with existing providers, develop policies and procedures, involve experienced providers capable of reaching rural physicians, accredit diverse local providers, restrict commercial entities as CME providers, and implement CME with proper preparation and phased implementation. These findings are likely to be very relevant for future work around further development of CME programs in Pakistan. The findings are also likely to be relevant to other countries with similar contexts and adds to our overall insight into the implementation of CME at a global level.

## Data Availability

The datasets used and/or analysed during the current study are available from the corresponding author on reasonable request.
